# Spontaneous closure of an isolated congenital perimembranous ventricular septal defect in two dogs

**DOI:** 10.1186/s12917-022-03266-9

**Published:** 2022-05-03

**Authors:** Anne van de Watering, Viktor Szatmári

**Affiliations:** Department of Clinical Sciences, Faculty of Veterinary Medicine, Utrecht University, Yalelaan 108, 3584 CM Utrecht, the Netherlands

**Keywords:** Screening, Natural history, Echocardiography, Puppies

## Abstract

**Background:**

Though spontaneous closure of isolated congenital ventricular septal defects in humans is very common, it has been rarely reported in dogs.

**Case presentation:**

A 4 month old Havanese dog and a 4.5 month old Chihuahua x Jack Russell terrier cross were presented for murmur evaluation to the authors’ institution. Both puppies were clinically healthy and had a loud systolic murmur on the right hemithorax. Echocardiography in both dogs revealed a small, isolated, restrictive perimembranous congenital ventricular septal defect. No echocardiographic signs of left ventricular volume overload or pulmonary hypertension were present. Re-check auscultation in both dogs revealed the absence of a murmur, and echocardiography showed no flow through the interventricular septum. In the 9 kg Havanese dog and the 4 kg mixed breed dog, spontaneous closure occurred at 13–17 months and 12–30 months, respectively.

**Conclusions:**

In both dogs the spontaneous closure of a congenital perimembranous ventricular septal defect took place in a young adult age. The mechanism of closure remains unclear.

## Background

Ventricular septal defect (VSD) is among the five most common congenital structural cardiac anomalies in dogs, and it is characterized by an opening in the interventricular septum, which allows shunting of blood from the left to the right ventricle during systole [1, 2]. Small and moderately sized defects typically cause a loud systolic murmur with the point of maximal intensity most often on the right hemithorax [3]. Though small defects do not cause morbidity or mortality, medium sized VSDs can lead to congestive left-sided heart failure through chronic left ventricular volume overload and/or pulmonary hypertension through increased pulmonary arterial blood flow [3, 4]. In dogs, VSDs are usually localized in the perimembranous part of the interventricular septum, directly underneath the aortic valve [3, 5]. Contrary to humans, muscular VSDs are rare in dogs [3, 5, 6]. While spontaneous closure of VSD, especially muscular VSDs, is very common in children [6], it has been rarely reported in dogs [7, 8].

The present report describes two dogs with spontaneous closure of a perimembranous VSD documented with cardiac auscultation and color Doppler echocardiography, both in a young adult age.

## Case presentation

### Case 1

**Fig. 1:**
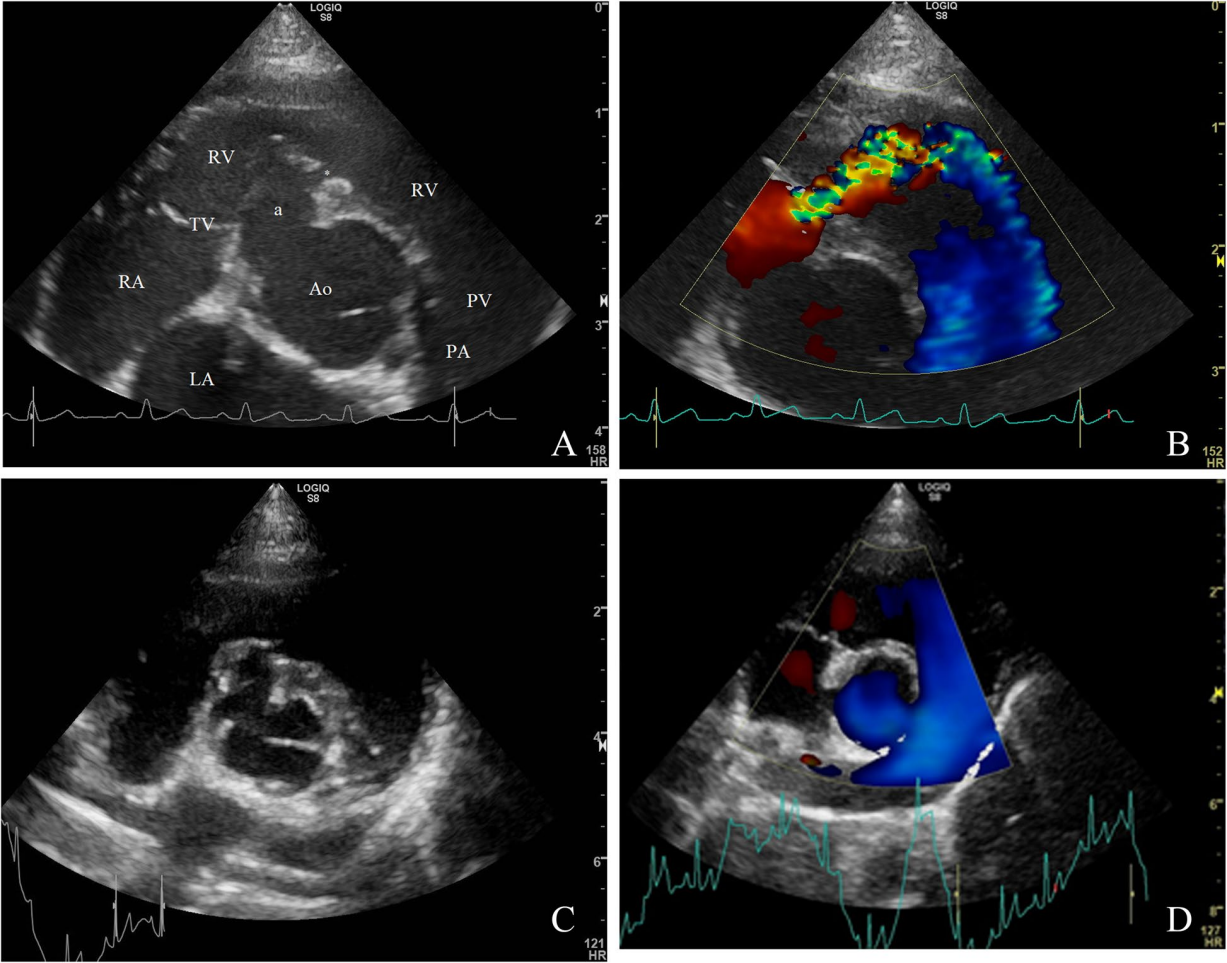
Standard right parasternal short-axis echocardiographic images (systolic frames at the level of the aortic valve) of the Havanese dog (case 1) at 4 months of age (**A**, **B**) and at 1 year and 10 months of age (**C**, **D**). **A** Two-dimensional image shows a membranous septal aneurysm adjacent to the aorta and the tricuspid valve. a = aneurysm of the membranous part of the interventricular septum, * = opening on the aneurysm, i.e., the actual ventricular septal defect, Ao = aorta, LA = left atrium, RA = right atrium, TV = tricuspid valve, RV = right ventricle, PV = pulmonic valve, PA = pulmonic artery trunk. **B** Color Doppler image shows a left-to-right shunting ventricular septal defect through the perforated septal aneurysm. The Nyquist limit was 80 cm/s in both directions. **C** Two-dimensional image showing the membranous septal aneurysm adjacent to the aorta and the tricuspid valve. **D** Color Doppler image showing the absence of flow through the membranous septal aneurysm. The Nyquist limit was 80 cm/s in both directions

A 4-month-old female intact, asymptomatic Havanese dog, weighing 4.1 kg was referred to the authors’ institution for evaluation of a cardiac murmur. At presentation the dog was bright, alert and responsive. Physical examination findings were unremarkable, except for the cardiac auscultation, which revealed a loud holosystolic murmur with the point of maximal intensity at the region of the tricuspid valve, with an intensity of 4 out of 6. Echocardiography showed a membranous ventricular septal aneurysm with a diameter of about 3.5 mm. This aneurysm had a small eccentric opening of about 1.2 mm, which allowed shunting of blood from the left ventricle into the right ventricular outflow tract. (Fig. 1A, B). The systolic peak interventricular Doppler-derived instantaneous pressure gradient using the simplified Bernoulli equation was 75 mmHg. The shunt opening on the right side of the septum was located immediately below the tricuspid valve. The left ventricular systolic and diastolic internal dimensions were within the reference ranges (the normalized left ventricular internal diameter was 1.55; reference < 1.7) [9, 10]. The left atrial size was normal with a left atrial to aortic ratio of 1.1 (reference < 1.6) measured on two-dimensional image according to the “Swedish method” [11]. No mitral valve regurgitation was present. The aortic valve showed a trivial regurgitation jet. Subjectively, the right atrial and right ventricular dimensions were normal and no abnormalities on the pulmonic trunk and the left and right pulmonary arteries were seen. There were trivial, assumed physiologic, tricuspid and pulmonic valve regurgitation jets noticed. There were no signs of pulmonary hypertension, such as flattening of the interventricular septum, and the pulmonary trunk diameter and its flow profile were normal [4]. The tricuspid and pulmonic valve regurgitation jets were too small to be able to measure their velocities. No other concomitant cardiac defect was found. The dog was discharged without any therapy and with a good long-term prognosis.

The referring veterinarian examined the dog at 13 months of age, when the dog was presented for routine vaccination. The murmur was present with an unchanged intensity compared to previous exams. However, 4 months later, at the age of 1 year and 5 months, when the dog was vaccinated again, the murmur was no longer heard by the referring veterinarian. Five months later, when the dog was 1 year and 10 months old, the dog was referred for a re-check echocardiography to the authors’ institution. According to the owners, the dog was still free of clinical signs. Physical examination was unremarkable and cardiac auscultation failed to reveal a murmur. The dog weighed 8.8 kg and had a body condition score of 4 out of 9. Echocardiography showed that the membranous septal aneurysm was still present but color Doppler echocardiography failed to show a detectable flow through the VSD (Fig. 1C, D). The left atrial and the left ventricular diastolic and systolic internal dimensions were within the reference ranges. The aortic valve and the aorta showed no abnormalities. The right atrium, right ventricle and pulmonary trunk appeared subjectively normal. The tricuspid and pulmonic valves continued to demonstrate a trivial, assumed physiologic, regurgitation jet.

### Case 2

**Fig. 2:**
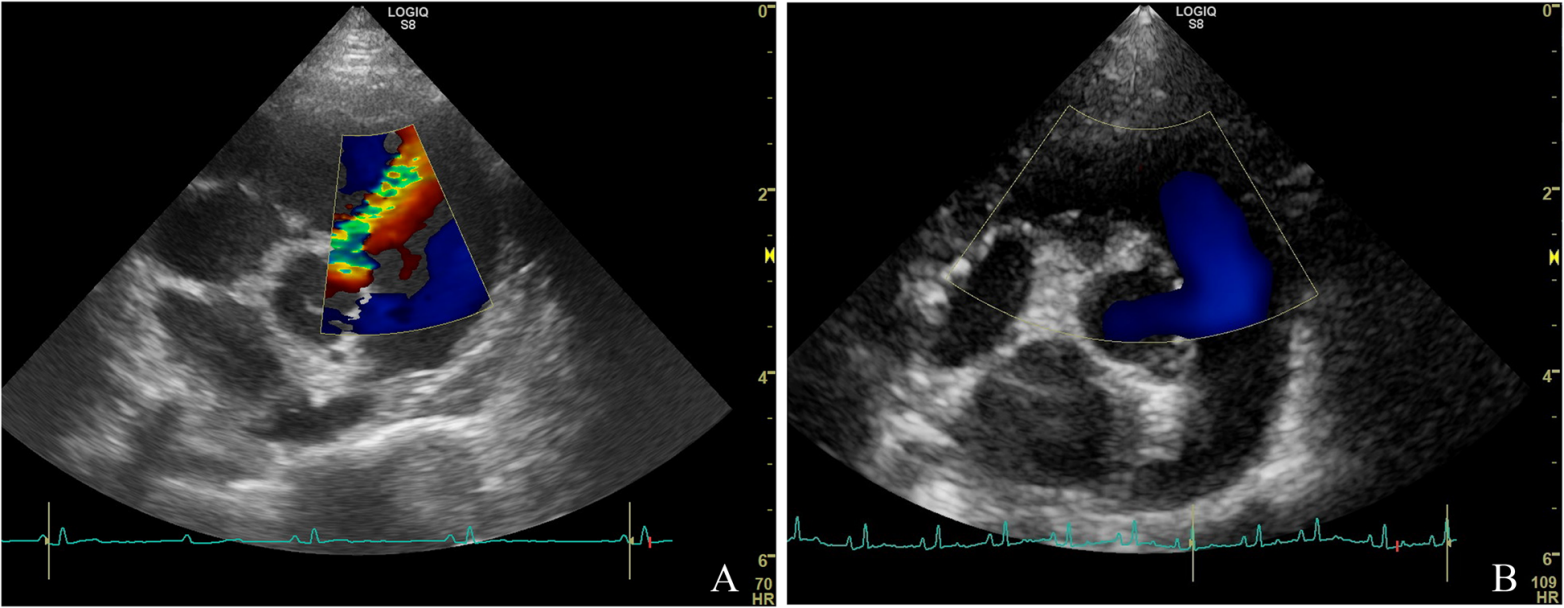
Standard right parasternal short-axis color Doppler echocardiographic images (systolic frames at the level of the aortic valve) of the mixed breed dog (case 2) at 4.5 months of age (**A**) and at 2.5 year of age (**B**). The Nyquist limit was 80 cm/s in both directions. **A** A left-to-right shunting ventricular septal defect is present adjacent to the aorta and the tricuspid valve. **B** Absence of flow through the previously detected ventricular septal defect can be appreciated

A 4.5-month-old female intact, asymptomatic crossbreed (Chihuahua x Jack Russell terrier) dog, weighing 3.0 kg was referred to the authors’ institution for evaluation of a cardiac murmur. At presentation the dog was bright, alert and responsive. Physical examination findings were unremarkable, except for cardiac auscultation, which revealed a loud systolic murmur with the point of maximal intensity at the region of the tricuspid valve, with an intensity of 5 out of 6. Echocardiography revealed left-to-right shunting VSD just below the aortic valve (Fig. 2A). The shunt opening on the right side of the septum was located immediately below the tricuspid valve. Unusually, the flow through the VSD could also be seen on two-dimensional images in the form of spontaneous echo contrast in the right ventricle. The systolic peak interventricular Doppler derived instantaneous pressure gradient using the simplified Bernoulli equation was 105 mmHg. The left ventricular systolic and diastolic internal dimensions were within the reference ranges (the normalized left ventricular internal diameter was 1.54; reference < 1.7) [9, 10]. Left atrial size was normal with a left atrial to aortic ratio of 1.5 measured according to the “Swedish method” [11]. No mitral valve regurgitation was present. The aortic valve showed mild regurgitation with the eccentric jet flowing through the VSD into the right ventricular lumen. Subjectively normal right ventricular and right atrial dimensions with no abnormalities on the pulmonic trunk and the pulmonic arteries were seen. There was no tricuspid valve regurgitation, but a mild, presumably physiologic, pulmonic valve regurgitation was noticed. No echocardiographic signs of pulmonary hypertension were seen [4]. No other concomitant cardiac defect was found. The dog was discharged without any therapy and with a good long-term prognosis.

Seven months later, at 1 year of age, the dog was presented for a follow up re-check examination at the cardiology service. The dog remained free of clinical signs according to the owner and weighed 4.1 kg with a body condition score of 4 out of 9. Physical examination findings were unremarkable, except for cardiac auscultation, which revealed a loud systolic murmur with similar intensity at the left and right cardiac base, with an intensity of 4 out of 6. Echocardiogram showed the presence of the previously diagnosed perimembranous VSD with a diameter of about 1.7 mm and a systolic pressure gradient between the ventricles of 115 mmHg. The mild aortic valve regurgitation was still present. The left ventricular diastolic internal diameter was still within the reference range (normalized left ventricular diastolic internal diameter of 1.43) [9, 10].

One and a half year later, at 2.5 years of age, the dog was presented for a pre-anesthetic evaluation of the heart because of a planned ovariectomy at the authors’ institution. The dog was still free of clinical signs. Physical examination findings were unremarkable, and cardiac auscultation revealed no murmur at this time. Echocardiography showed no abnormalities, other than a trivial mitral valve insufficiency. The perimembranous VSD was no longer visible with two-dimensional and color Doppler echocardiographic modes (Fig. 2B). The left ventricular diastolic internal diameter was unchanged (normalized left ventricular internal diameter of 1.40) and no echocardiographic signs of pulmonary hypertension were noticed [4, 9, 10].

## Discussion and conclusions

The present case series describes the spontaneous closure of an isolated congenital small restrictive perimembranous ventricular septal defect in two small breed dogs beyond 1 year of age. In the first case, the peak pressure gradient was 75 mmHg, which is lower than the value that defines a VSD to be restrictive (> 80 mmHg). In the absence of increased right ventricular systolic pressure, this value is most likely the result of underestimation of the systolic peak flow velocity because of a suboptimal Doppler angle. In the veterinary literature there are only two case reports describing a total of three dogs where a spontaneous closure of a ventricular septal defect took place [7, 8]. In Breznock’s report, in both dogs, the closure of the VSD was documented with physical examination and angiocardiography [8]. Both dogs were small breeds (a toy poodle and a miniature Schnauzer cross) and both of them underwent a successful surgical ligation of a concomitant left-to-right shunting patent ductus arteriosus at 8 weeks of age [8]. In these dogs, the murmur could not be detected at 12 and 16 months of age, respectively [8]. The other case report describes that a small VSD in a 5-month-old female Maltese dog, diagnosed with auscultation and color Doppler echocardiography, was spontaneously closed at 1 year of age, which is similar to the two cases described in this paper [7].

Spontaneous closure occurs frequently in humans, with higher incidence of muscular defects compared to perimembranous VSDs [6, 12–15]. Besides the anatomical localization, the size of the defect is an important factor, as smaller defects tend to close more often [6, 12]. In all the reported dogs, the defects were smaller than 2 mm [7, 8]. In humans, various mechanisms are described for spontaneous closure of VSDs, depending on the anatomical localization of the defect [12, 13, 15]. While closure of muscular VSDs are thought to be caused by the growth of the surrounding septum and fibrous tissue formation, perimembranous VSDs are suspected to be occluded as a result of right ventricular jet lesions, adherence of the tricuspid valve leaflets, aneurysm of the membranous septum and deposition of fibrin over the margins of the defect [12]. A membranous aneurysm may be a prelude to the spontaneous closing of small ventricular septal defects, where endocardial proliferation or hypertrophy appears around the defect on the right, like an aneurysmal protrusion of the membranous septum [14]. In addition to these mechanisms, in the case of perimembranous VSDs, when the defect is in close proximity to the tricuspid and aortic valves, reduplication of the tricuspid valve tissue and prolapse of an aortic valve leaflet have been described as the cause of spontaneous VSD closure [12]. The way in which turbulent blood flow results in a jet lesion and consequently a proliferation of endocardial fibrous tissue is similar to the mechanism described in the pathogenesis of double-chambered right ventricle and discrete subaortic stenosis [16]. The cause of closure of the VSDs in the present report cannot be known as both dogs remained alive and post-mortem examination has yet to be performed.

In humans, age appears to have a significant influence on the incidence of spontaneous VSD closure, while gender is less likely to affect this process [12–15]. Although spontaneous VSD closure can occur at any age in humans, it usually occurs during the first year of life or shortly thereafter [12, 17]. Interestingly similar to human patients, the age of VSD closure in all the five reported dogs (three from previously published reports and the two dogs from the present paper), took place shortly after 1 year of age [7, 8]. One possible explanation may be that the suspected endocardial fibrous proliferative changes do not occur until after the dogs have reached adult size and are no longer growing. Another possible explanation could be that proliferation starts early in life but takes more than a year to reach the extent which will close the VSD .

Both dogs in our report were intact females. One dog in Breznock’s case report was male and the sex of the other one was not reported [11]. The Maltese dog from the single case report was female [7]. Whether the seemingly female predisposition for VSD in the reported five dogs is a coincidence or if sex does influence this process, remains unknown. In human medicine, separate studies report conflicting gender predispositions [12, 18].

As both dogs in Breznock’s case report underwent surgical ligation of a left-to-right shunting patent ductus arteriosus several months before spontaneous closure of the VSD took place, the role of this surgery was discussed as a potential contributing factor [11]. Neither dogs in our case report had undergone a surgery of any kind or a general anesthesia before closure of the VSD, therefore a possible contribution of such a procedure remains impossible.

Disappearance of the murmur in a dog where previously a left-to-right shunting VSD was diagnosed can also result from the development of pulmonary hypertension (Eisenmenger’s syndrome), where the left and right ventricular systolic pressures equalize [3, 4, 19]. Performing an echocardiogram is therefore an important diagnostic test to identify the cause of murmur disappearance.

It is possible that VSDs in dogs close more often than is currently thought. One of the possible reasons why spontaneous closure of VSDs in dogs has not been more frequently reported may be the lack of serial cardiac auscultations and echocardiographic re-check examinations in these dogs. Small VSDs are known to have a good long-term prognosis that is presumably why regular monitoring of these dogs does not routinely take place.

In conclusion, the present report describes two female dogs, in which a congenital isolated small, restrictive perimembranous ventricular septal defect closed spontaneously shortly after 1 year of age.

## Data Availability

Not applicable.

## Abbreviation

VSD: Ventricular septal defect
